# Second-harmonic optical vortex conversion from WS_2_ monolayer

**DOI:** 10.1038/s41598-019-45424-4

**Published:** 2019-06-19

**Authors:** Arindam Dasgupta, Jie Gao, Xiaodong Yang

**Affiliations:** Department of Mechanical and Aerospace Engineering, Missouri University of Science and Technology, Rolla, MO 65409 USA

**Keywords:** Integrated optics, Two-dimensional materials

## Abstract

Wavelength, polarization and orbital angular momentum of light are important degrees of freedom for processing and encoding information in optical communication. Over the years, the generation and conversion of orbital angular momentum in nonlinear optical media has found many novel applications in the context of optical communication and quantum information processing. With that hindsight, here orbital angular momentum conversion of optical vortices through second-harmonic generation from only one atomically thin WS_2_ monolayer is demonstrated at room temperature. Moreover, it is shown that the valley-contrasting physics associated with the nonlinear optical selection rule in WS_2_ monolayer precisely determines the output circular polarization state of the generated second-harmonic vortex. These results pave the way for building future miniaturized valleytronic devices with atomic-scale thickness for many applications such as chiral photon emission, nonlinear beam generation, optoelectronics, and quantum computing.

## Introduction

Orbital angular momentum (OAM) and spin angular momentum (SAM) of photons have emerged as important degrees of freedom to store, control and transport information using light^1–3^. SAM represents the circular polarization state of light and OAM is intrinsically related to the azimuthal dependence of optical phase. An optical vortex beam possessing the helical phase structure of exp*(ilϕ*) carries the OAM of *l*ℏ per photon and exhibit the doughnut-shaped intensity profile^4^, where *l* is the topological charge (TC) and *ϕ* is the azimuthal angle around the phase singularity. As *l* is an unbounded number, optical vortex beams provide accessibility to infinite orthogonal states of OAM and thus serve as unmatched optical carriers for large-scale information transfer^5–7^. Due to this fact, optical vortices have been studied extensively in the context of processing and encoding information in optical communication and quantum computing^8–10^. Moreover, optical vortices have been harnessed at micron to sub-micron scales for realizing on-chip applications such as quantum memory devices^11^, nano-optical tweezers^12–16^ and many more.

On the other hand, harmonic generation and spontaneous parametric down-conversion have been incorporated for building nonlinear optical switches and wavelength converters in all-optical wavelength-division multiplexing networks and quantum information processing. Therefore, the efficient conversion and control of OAM and SAM for the second-harmonic generation (SHG) signal adds more flexibility and dimensions in optical communication^1,17–20^. Until now, nonlinear crystals are mostly used for the generation of second-harmonic (SH) optical vortex beams^20–23^. It has been demonstrated that the TC of the SH vortex beam depends on the TC of the pump laser beam^23^. Furthermore, the circular polarization state of the SH vortex is inherently determined by the rotational symmetry of the crystal^24^, but the presence of crystal defects, deformation and scattering from optical phonons often deteriorate the degree of circular polarization of the SH signals. In addition, the bulky size of nonlinear crystal also hinders its on-chip integration.

**Figure 1:**
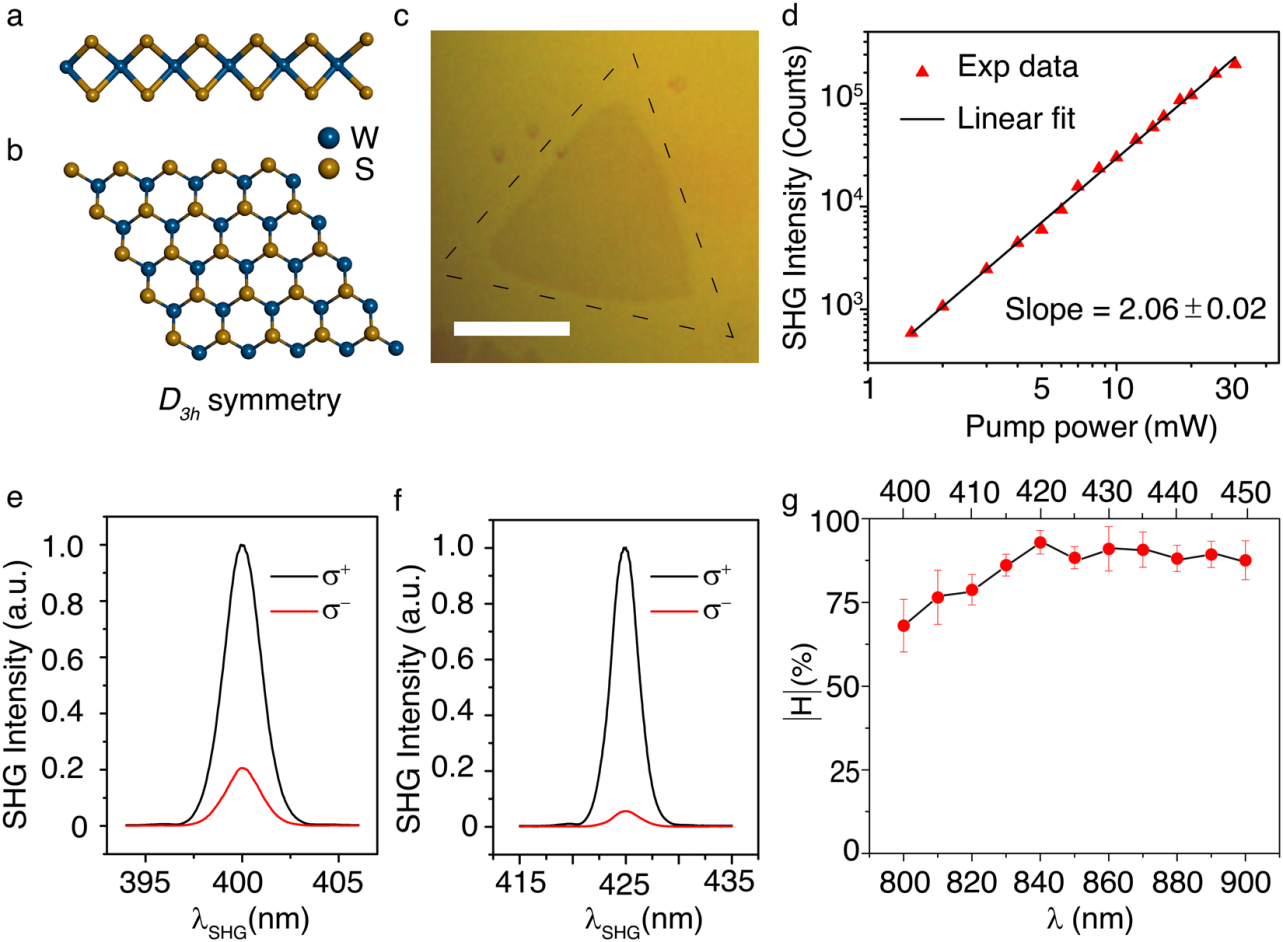
Valley-dependent circular polarization SHG selection rule off the exciton resonance. (**a**,b) Schematics of the side view and top view of the crystal structure for WS_2_ monolayer. (**c**) Optical transmission microscope image of an isolated WS_2_ monolayer triangular flake. Scale bar is 20 μm. (**d**) Double log-scale plot of the measured SHG intensity from WS_2_ monolayer as a function of the incident pump power under σ^−^ excitation at 800 nm. (**e**,f) Circular polarization-resolved SHG spectra from the WS_2_ monolayer under σ^−^ excitation at 800 nm and 850 nm. (**g**) Evolution of the SHG helicity as a function of the fundamental wavelength under σ^−^ excitation. Bottom horizontal scale indicates the fundamental wavelength while the top horizontal scale indicates the corresponding SHG wavelength.

In that perspective, SHG from atomically thin transition metal dichalcogenide (TMDC) monolayer has attracted a lot of attention in the recent past, as the TMDC monolayer can be easily interfaced at nanoscale and exhibits very high second-order nonlinear susceptibility *χ*^*(2)*^ with the absolute value of few nm/V, orders of magnitude higher than common nonlinear crystals^25–27^. Along with inversion symmetry breaking, the three-fold rotational symmetry (D_3*h*_) in hexagonal lattice (Fig. 1a,b) and strong spin-orbit coupling in TMDC monolayers result in the presence of energy-degenerate valleys in momentum space at the corners of the first Brillouin zone (*K* and *K*′ points). Valley is recognized as an extra degree of freedom (in addition to spin and charge) for electrons to store or carry information in the modern field of valleytronics^28^. The inequivalent valleys (*K* and *K*′) in TMDC monolayers are composed of opposite spin states and thus can selectively absorb or emit the right-handed (*σ*^+^) or left-handed (σ^−^) circularly polarized light^29–31^. The nonlinear optical selection rule in TMDC monolayers has been studied where counter-circular SH photons are produced due to the *D*_3*h*_ crystal symmetry^32–34^. Therefore, spin-selective SHG in TMDCs can be utilized as an optical readout for valleytronic memory devices^29,35,36^. It has also been demonstrated that unlike conventional nonlinear crystals, such SHG selection rule in TMDC monolayers is immune to impurity, defects and phonon scattering due to the large separation between the band edges in momentum space^34^. For practical applications, it will be exciting to explore the nonlinear optical beam generation and conversion process from a TMDC monolayer at room temperature.

In this work, it is demonstrated that the valley-dependent spin selection rule of SHG in a tungsten disulfide (WS_2_) monolayer remains almost intact off the exciton resonance with high SHG helicity at room temperature. The spin flipping and OAM doubling of the generated SH optical vortex beams from only one WS_2_ monolayer under the excitation of fundamental vortex beams with different TCs are also observed. Our results can be harnessed to realize efficient wavelength- and OAM-based multiplexing, demultiplexing and encoding archetypes for building miniaturized atomic-scale communication and computing devices. The results also provide new opportunities for advancing 2D materials-based valleytronic devices such as chiral light emitters, nonlinear beam converters, optoelectronic and quantum chips.

## Results

### Valley-dependent SHG selection rule at room temperature

The valley-dependent circular polarization SHG selection rule arises from the crystal rotational symmetry requirement for the conservation of total angular momentum during the photon-lattice interaction. In conventional nonlinear crystals with *C*_*3*_ rotational symmetry, a spin-flipped SHG is generally observed where the lattice supplies the extra angular momentum to the SH photons. However, in TMDC monolayers, for two-photon fundamental excitation above the bandgap, valley-contrasting physics dictates the SHG selection rule by introducing the valley angular momentum (VAM), which can be written as^32,33^

1 $$\Delta m\hslash =\Delta {\rm{\tau }}\hslash +3{\rm{N}}\hslash ,({\rm{N}}=\pm 1)$$

where $\Delta m\hslash$ is the change in the SAM of the photons, and $\Delta \tau \hslash$ is the change in the VAM. The term $3N\hslash$ represents the change in the angular momentum of the crystal lattice due to the three-fold rotational symmetry (Fig. 1b). The local atomic orbital angular momentum and opposite Berry curvatures of *K* and *K*′ valleys give rise to the presence of an out-of-plane component of VAM being $\tau \hslash =-1\hslash$ or $+1\hslash$ in the conduction band and $\tau \hslash =0\hslash$ in the valence band for *K* or *K*′ valleys, respectively. Therefore, electronic transition from the valence band to the conduction band in *K* or *K*′ valleys requires $-1\hslash$ or $+1\hslash$ change in the angular momentum. By considering the SHG process under *σ*^+^ excitation, the absorption of two fundamental photons in *K* valley causes the photon SAM change of $+2\hslash$ and the excess angular momentum of $+3\hslash$ in the crystal lattice. Therefore, the emission of one SH photon with the flipped spin (σ^−^) becomes inevitable. Similarly, under time reversal, the absorption of two σ^−^ fundamental photons at *K*′ valley will result in the emission of one *σ*^+^ SH photon.

As one typical TMDC material, single-crystal WS_2_ monolayer triangles grown by the low-pressure chemical vapor deposition method on a c-cut (0001) sapphire substrate (2D Semiconductors) are considered in the experiment. Figure 1c shows an optical transmission microscope image of an isolated WS_2_ monolayer triangular flake. In addition to the large valance band spin splitting (0.426–0.433 eV)^37–39^ which makes the valley-contrasting physics more robust, WS_2_ monolayer possesses large *χ*^(2)^ in the 800–900 nm wavelength range^40–42^. Figure 1d plots a log-log variation of the collected SHG signal as a function of the incident pump power under σ^−^ excitation at the fundamental wavelength of 800 nm. The power scaling of the SHG follows the expected quadratic dependence very well. According to Fig. 1e,f, the circular polarization-resolved SHG spectra at room temperature from the WS_2_ monolayer under σ^−^ excitation at the fundamental wavelengths of 800 nm and 850 nm reveal that most of the SHG signal is spin flipped with σ^+^ polarization. The helicity of the circularly-polarized SHG signal is defined as $H=\frac{I({\sigma }^{-})-I({\sigma }^{+})}{I({\sigma }^{-})+I({\sigma }^{+})}\times 100 \%$ where *I*(σ^−^) and *I*(σ^+^) are the measured SHG intensity of the σ^−^ and σ^+^ components. The measured *H* is approximately −70% and −90% at the fundamental wavelengths of 800 nm and 850 nm, respectively. The negative value of *H* indicates that the two σ^−^ fundamental photons are simultaneously absorbed at K′ valley to produce one counter-circular σ^+^ SHG photon. Figure 1g shows the variation of SHG helicity as a function of the SHG wavelength from 400 to 450 nm which is away from the exciton resonance of WS_2_ monolayer (around 620 nm), where the data are averaged over the measurements from six samples. The less than 10% error bars in the measured helicity values signify that all the samples exhibit more or less similar behavior, so that the sample-to-sample variability of the SHG response is quite low. The measured helicity values increase monotonically from −70% to −94% in the SHG wavelength range of 400–420 nm, while high values larger than −90% are observed in 420–450 nm. It has been demonstrated that the elevated intravalley scattering and intervalley scattering at room temperature and high-energy excitation will significantly deteriorate the valley-dependent optical spin selection in both cases of one-photon and two-photon photoluminescence^33,34,43,44^. While our results reestablish that SHG process occurs before the intervalley scattering of carriers^34^. SHG is an instantaneous process with ultrashort-lived participating virtual states, with the signal only generated during the femtosecond laser pulse excitation of the WS_2_ crystals. At room temperature, the intervalley scattering lifetime is shortened to picosecond timescale^43^, but it is still much longer than the lifetime of the participating virtual states in the SHG process, and thus the SHG response is almost free of the intervalley scattering process. Therefore, the SHG helicity is not affected by the significantly increased intravalley and intervalley scattering of the excited electrons at room temperature. The helicity is rather largely dependent on the initial absorption condition imposed by the valley selection of the circularly polarized light. As a result, valley-dependent circular polarization SHG selection rule for WS_2_ monolayer is still intact at room temperature. The relatively low SHG helicity in the wavelength range of 400–420 nm shown in Fig. 1g may be related to the presence of disorders or deformation in the crystal lattice, which tends to weaken the valley-dependent selection rule far away from the exciton resonance wavelength of WS_2_ monolayer around 620 nm^45^.

### Second-harmonic vortex conversion

**Figure 2:**
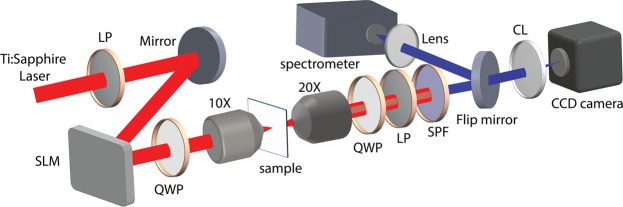
Schematic diagram of the experimental setup. (LP: linear polarizer; SLM: spatial light modulator; QWP: quarter-wave plate; CL: cylindrical lens; SF: spectral filter).

Next, valley-dependent SH optical vortex conversion from WS_2_ monolayer is investigated by exciting the monolayer with an OAM-carrying fundamental vortex beam under circular polarization. Figure 2 shows the schematic of the experimental setup. The femtosecond laser pulse at the fundamental wavelength from a tunable ultrafast Ti:Sapphire oscillator (Coherent Chameleon, wavelength range 690–1040 nm, repetition rate 80 MHz, pulse width <100 fs) is projected onto a spatial light modulator (SLM, Holoeye) to form the desired vortex phase profile. The linear polarization of the laser beam is set along the long axis of the SLM and the beam is expanded to match the SLM screen size. Upon reflection from the SLM, different Laguerre-Gaussian (LG) modes with TC = 1, 2 and 3 are generated. A quarter-wave plate is used to transform the linear polarization of vortex beam into circular polarization which is focused on the WS_2_ monolayer through a 10X objective lens. The transmitted fundamental and SHG signals are then collected with another 20X objective lens and guided towards a color charge-coupled device (CCD) camera or a spectrometer. The images of the fundamental or SH vortex are captured with the color CCD camera by removing or inserting a band-pass filter. On the other hand, the spectrum and intensity of the SH vortex are analyzed by using the spectrometer (Horiba, iHR 550) where a short-pass filter in the path is used to block the fundamental excitation. The TCs of the fundamental vortex and SH vortex are determined by counting the dark stripes in the captured image patterns on the CCD camera focused by a cylindrical lens^46^. A cylindrical lens squeezes light in one direction (power axis) without affecting the light in the perpendicular direction (plano axis) and therefore induces strong astigmatism in the optical beam. Here, the cylindrical lens is used to perform the astigmatic transformation of an optical vortex beam into a strongly deformed vortex core in the focal plane. During the astigmatic transformation, the high-TC optical vortex splits into its constituent elementary vortices with unity TC which are strongly deformed similar to edge dislocations to form an extended pattern of tilted dark stripes near the focal plane of the cylindrical lens. Therefore, the number of the dark stripes represents the vortex TC and the stripe orientation indicates the charge sign. The circular polarization state of the SH vortex is analyzed with the combination of a quarter-wave plate and a linear polarizer right after the collection objective lens.

**Figure 3:**
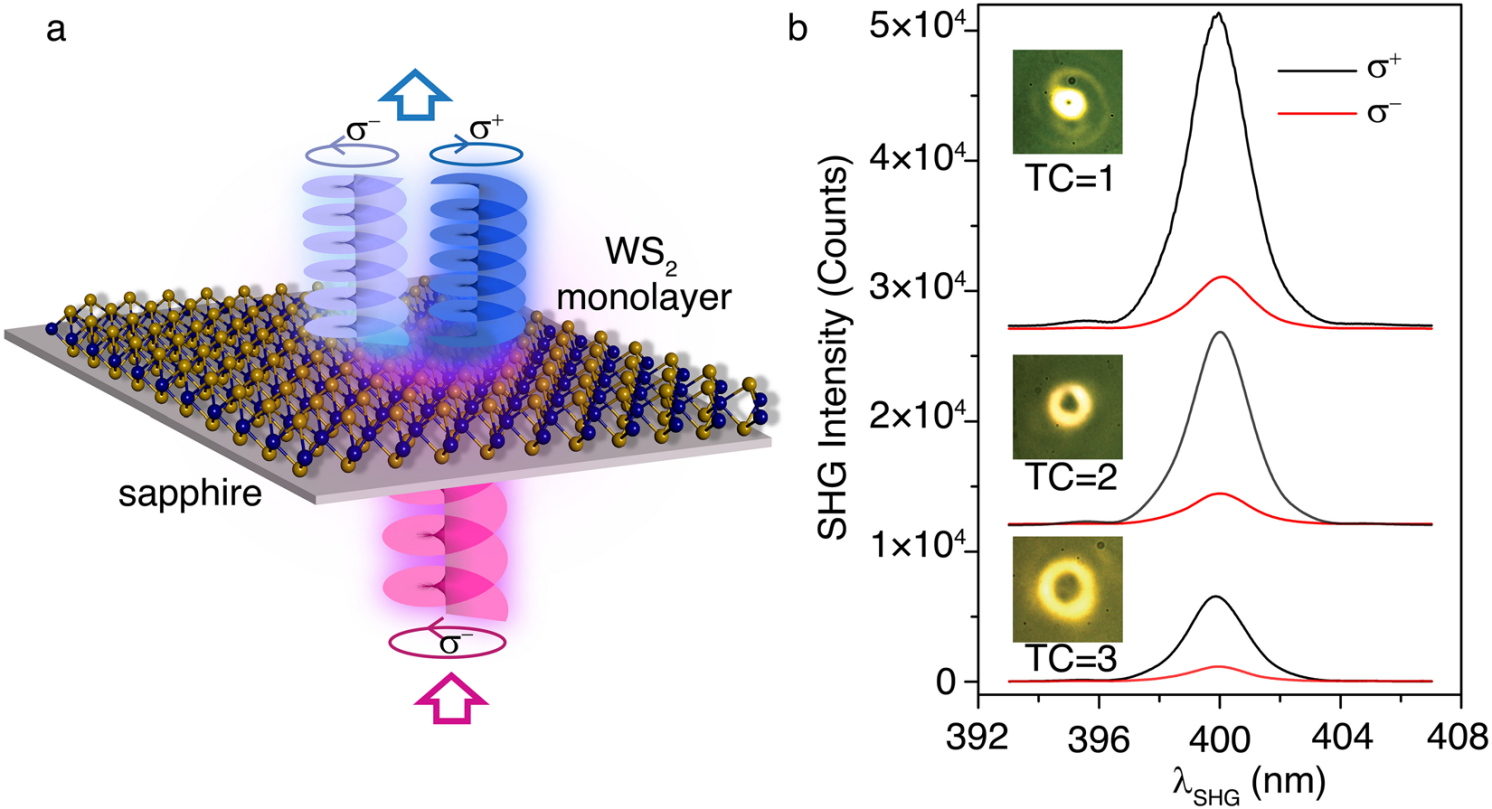
Valley-dependent circular polarization SHG selection rule for optical vortex excitation. (**a**) Schematic illustration of the experiment. (**b**) Circular polarization-resolved SHG spectra from WS_2_ monolayer under the excitation of σ^−^ fundamental optical vortices with TCs 1, 2 and 3 at 800 nm. Inset for each spectrum is an optical image showing the doughnut-shaped intensity profile of the fundamental optical vortex.

As illustrated in Fig. 3a, the circular polarization-resolved SHG spectroscopy and imaging measurements are performed from WS_2_ monolayer under the excitation of circularly-polarized fundamental vortex beam. The fundamental wavelength at 800 nm is purposely selected with the lowest SHG helicity (Fig. 1e), which enables the possibility for examining the SH vortex evolution of both the co-polarized and counter-polarized components. Before measuring the OAM conversion, we recheck whether the valley-dependent SHG selection rule is still intact under optical vortex illumination. Figure 3b shows the circular polarization-resolved SHG spectra from WS_2_ monolayer, illuminated with σ^−^ fundamental optical vortices with TCs of 1, 2 and 3. The insets are the optical images of the doughnut-shaped intensity patterns of the incident fundamental vortex beams. The impinging pump power is set as a constant of 10 mW throughout the measurement. As the TC value increases, the radius of the doughnut pattern increases, leading to the reduced pump power and the inevitable decrease of SHG intensity. Similar to the case under Gaussian beam excitation with the measured SHG helicity of around −70% (Fig. 1e), a high conversion to σ^+^ SH signal is also observed under optical vortex excitation, where the SHG helicity maintains to be −71.18%, −70.56% and −70.68% for the fundamental optical vortices with TCs of 1, 2 and 3, respectively.

**Figure 4:**
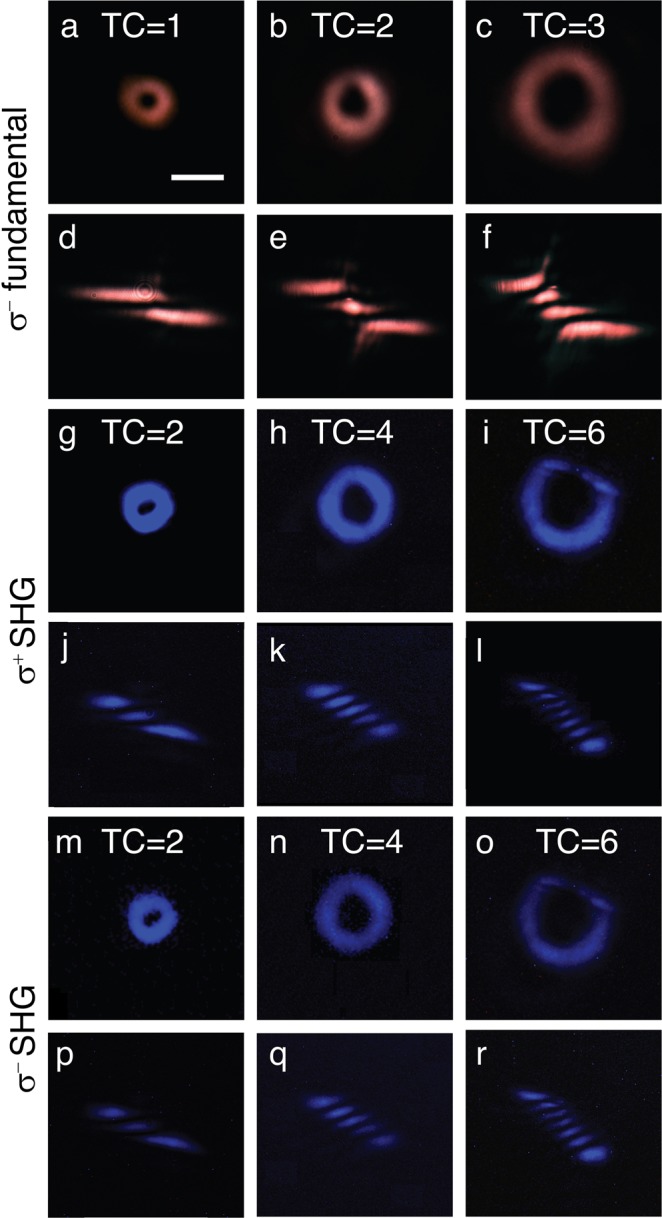
Second-harmonic vortex conversion from WS_2_ monolayer. (**a–c**) Color CCD images of the doughnut-shaped intensity patterns of the σ^−^ fundamental vortex beams with TCs of 1, 2 and 3. (**d–f**) Corresponding TC images of the fundamental vortex beams. (**g–i**) Color CCD images of the doughnut-shaped intensity profiles of the σ^+^ SH vortex beams from WS_2_ monolayer. (**j–l**) Corresponding TC images of the σ^+^ SH vortex beams. (**m–o**) and (**p–r**) are the color CCD images and TC images of the σ^−^ SH vortex beams. Scale bar is 10 μm.

**Figure 5:**
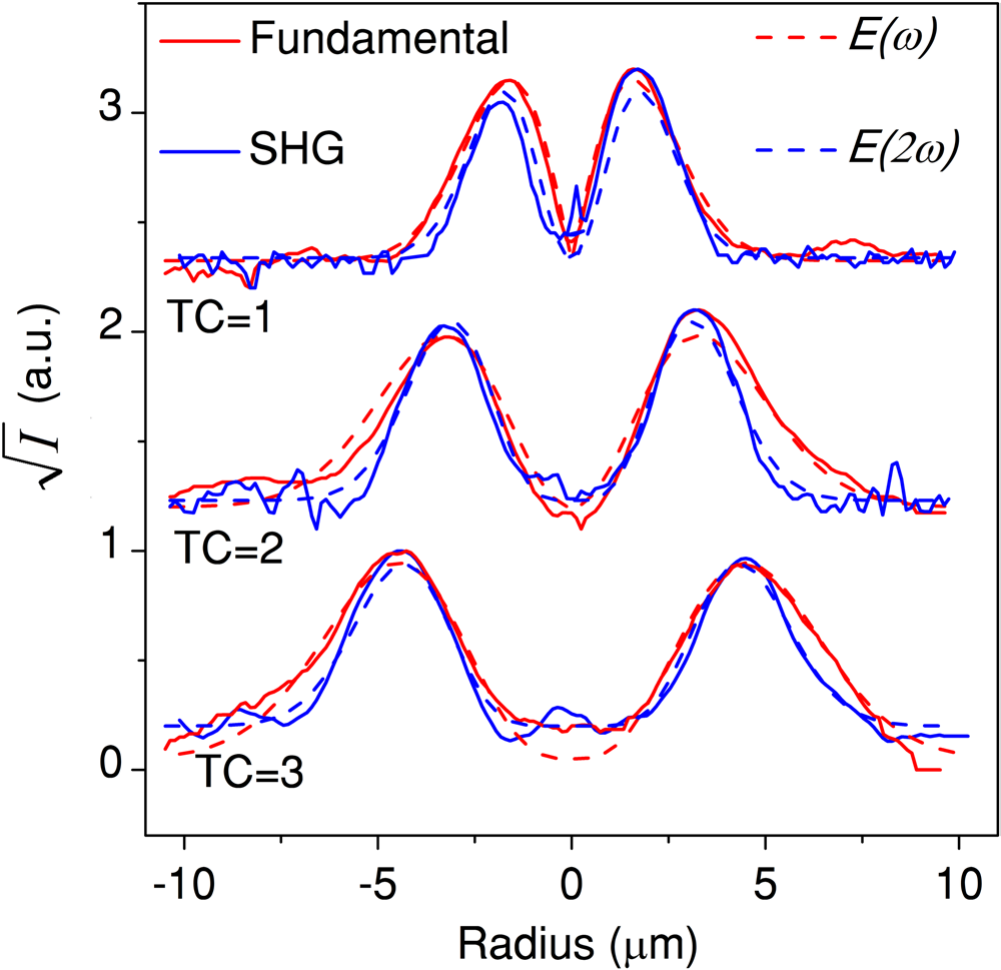
Comparison of the beam sizes of the fundamental and SH converted vortex beams. Measured line profiles of the square root of intensity for the fundamental and SH vortex beams for three fundamental vortex excitations with TC = 1, 2 and 3 (solid curves), fitted by the calculated electric field profiles of ${\boldsymbol{E}}({\boldsymbol{\omega }})$ and ${\boldsymbol{E}}(2{\boldsymbol{\omega }})$ (dashed curves).

The OAM conversion through SHG process from WS_2_ monolayer is then characterized under the excitation of σ^−^ fundamental optical vortex. Figure 4a,b,c are the recorded transmission images of the doughnut-shaped intensity patterns of the fundamental vortex beams with TCs of 1, 2 and 3 focused on the WS_2_ monolayer. The associated TC for each fundamental vortex is confirmed by counting the dark strips in the cylindrical lens converted image (Fig. 4d–f). The circular polarization-resolved SH vortex beams are also monitored by the CCD camera by blocking the fundamental beam using a 400 nm band-pass filter. Figure 4g–i are the recorded images of the counter-polarized (σ^+^) SH vortex components under the excitation of the three σ^−^ fundamental optical vortex beams. The TCs of the converted SH vortex beams are presented in Fig. 4j–l, indicating the doubled OAM carried by the SH vortex with the TCs of 2, 4 and 6, respectively. In order to obtain comparable image contrast, gradually longer exposure time in the CCD camera is used for capturing the image of the higher-order SH vortex. Figure 4m–o are the captured images for the co-polarized (σ^−^) SH vortex components, while Fig. 4p–r also show the TC doubling. Since the co-polarized SHG signal is very weak, the integration time of CCD camera is set as five times of that for capturing the counter-polarized images. The TC doubling in SHG process is a well-known phenomenon in nonlinear optics originating from the conservation of OAM^23^. If the electric field of the fundamental beam at frequency *ω* is given by $E(\omega )$, then the electric field of the SH beam $E(2\omega )$ is proportional to *E*(*ω*) by $E(2\omega )\propto E{(\omega )}^{2}$. A LG beam carrying the TC of *l*_1_ without any radial component is expressed as $E(\omega )={E}_{1}\,\exp [-(\frac{{r}^{2}}{{w}_{1}^{2}}+i{l}_{1}\phi )]{(\frac{r\sqrt{2}}{{w}_{1}})}^{{l}_{1}}$, where *r* is the distance from beam center, *w*_1_ is the radius for which the Gaussian term falls to 1/*e* of its on-axis value. Therefore, the SH LG mode with $E(2\omega )$ proportional to $E{(\omega )}^{2}\,\,$takes the following form $E(2\omega )={E}_{1}^{2}\,\exp [-(\frac{{r}^{2}}{{w}_{2}^{2}}+i{l}_{2}\phi )]{(\frac{r\sqrt{2}}{{w}_{1}})}^{{l}_{2}}$, with a doubled TC ${l}_{2}=2{l}_{1}$ and ${w}_{2}$ reduced by a factor of $\sqrt{2}$ from ${w}_{1}$. Figure 5 plots the measured line profiles of the square root of intensity for the fundamental and the corresponding SH converted vortex beams with different TCs, which are fitted by the calculated electric field profiles of $E(\omega )$ and $E(2\omega )$. It shows that the measured ${w}_{2}/{w}_{1}$ ratio is 0.79, 0.71 and 0.68 for the cases of TC = 1, 2 and 3 fundamental vortex excitation, respectively, which is close to 1/$\sqrt{2}$.

## Discussion

In summary, we have demonstrated the SH optical vortex generation from WS_2_ monolayer under the excitation of circularly polarized fundamental vortex beam, with the simultaneous conversion of wavelength, SAM and OAM of photons through the SHG process. By performing the circular polarization-resolved SHG spectroscopy, we observe that the valley-dependent circular polarization SHG selection rule remains almost intact off the exciton resonance at room temperature and it is not affected by intervalley scattering processes. Furthermore, we show that the OAM of the converted SH vortex beam gets doubled compared to that of the fundamental vortex beam. The demonstrated parallel and efficient control over the wavelength, SAM and OAM of photons with TMDC monolayers can be exploited for multiplexing and encoding information in optical communication and quantum information processing. Also, the valley-dependent SHG selection rule along with the OAM conversion can be harnessed for OAM-multiplexed optical read-out channels in TMDC valleytronic memory devices. Additionally, the atomic-scale thickness of TMDC monolayer facilitates effective subwavelength interfacing for on-chip applications in photonic integrated circuits and quantum processors. Our results also create many possibilities for realizing future 2D materials-based valleytronic devices such as chiral light emitters, nonlinear beam generators, photodetectors and quantum computing chips.
